# Composition, diversity and functional potential of bacterial community in four stony coral species from the South China Sea

**DOI:** 10.3389/fmicb.2026.1759094

**Published:** 2026-02-10

**Authors:** Zimu Li, Xinyu Liao, Li Mo, Qi Liao, Kaijun Lin, Xinyi Bao, Jijia Sun, Xiaoyong Zhang

**Affiliations:** University Joint Laboratory of Guangdong Province, Hong Kong and Macao Region on Marine Bioresource Conservation and Exploitation, College of Marine Sciences, South China Agricultural University, Guangzhou, China

**Keywords:** bacterial community structure diversity, coral symbiotic bacteria, functional prediction, high-throughput sequencing, South China Sea stony corals

## Abstract

Recent investigations of coral symbiotic microorganisms have largely centered on their ecological functions, while systematic analyses of the community composition, diversity, and functional potential of bacteria associated with different coral species remain limited. This study presents the first systematic analysis of the distinct community structures and highly conserved core functions of symbiotic bacteria in four species of stony corals *Favites abdita* (*Fa*), *Favia speciosa* (*Fs*), *Montipora digitata* (*Md*), and *Porites solida* (*Ps*) from the South China Sea by high-throughput sequencing. The results identified 23 phyla and 250 genera of bacterial taxa, revealing considerable taxonomic richness in these coral-associated bacterial communities. Significant differences (*p* < 0.05) in bacterial community composition were observed among four coral species. Proteobacteria was the absolutely dominant phylum in *Fa*, *Fs*, and *Ps*, whereas *Md* was dominated by the phylum Firmicutes. At the genus level, the core microbial communities of the four coral species were similar in composition but exhibited marked differences in abundance. *Md* showed the highest species richness and diversity, and *Fs* the lowest. Among them, the *Fa*, *Fs*, and *Ps* groups were dominated by *Ruegeria*, while the *Md* group was characterized by a high abundance of *Paramaledivibacter*, which was significantly more abundant than in other groups. Functional prediction indicated that the relative abundances of core functional categories, such as amino acid transport and metabolism and energy production and conversion, were highly consistent across the four coral species, reflecting functional conservation within these communities. These findings enrich the basic data on the diversity and function of Coral symbiotic microorganisms in the South China Sea, revealing the connection between coral community variability and the conservation of core functions.

## 1 Introduction

Coral reef ecosystems, often termed the “rainforests of the sea,” are among the most productive and biologically diverse systems on Earth (Chen et al., 2015; Sun et al., 2023). As a cornerstone of global marine biodiversity, they represent a vital resource for human survival. Although they cover merely 0.2% of the seafloor, coral reefs provide habitat and food sources for at least 25% of marine species (Anthony, 2016; Hill et al., 2021). Coral reefs contribute an estimated $375 billion annually to the global economy (Costanza et al., 1998; Rosenberg et al., 2007), primarily through fisheries, tourism, and coastal protection. Additionally, coral reefs and their symbiotic microorganisms are rich sources of bioactive compounds Natural products serve as a cornerstone of anticancer drug discovery, with over 60% of current agents originating from or inspired by natural sources. The marine realm in particular has emerged as a rich reservoir of unique and potent compounds with significant therapeutic potential (Alves et al., 2018; Bandaru et al., 2025). Coral microbial communities also play essential roles in nutrient cycling (Wegley et al., 2007) and respond rapidly to environmental disturbances (Bourne et al., 2016).

Coral symbiotic microorganisms are indispensable components throughout the coral life cycle. Their symbiotic relationship with corals directly influences coral survival, growth, and environmental adaptability (Krediet et al., 2013). These symbiotic microorganisms primarily include bacteria, fungi, and microalgae. Among them, bacterial communities are fundamentally intertwined with corals as essential symbionts (Bernasconi et al., 2019). This coral-associated bacteria play a vital role in nutrient cycling and metabolic processes, serving as the core safeguard for maintaining coral health (Su et al., 2020). This close symbiosis means that when the marine environment changes, corals can adapt to environmental fluctuations by regulating their interactions with symbiotic microorganisms (Macknight et al., 2021; Zhao et al., 2022). These bacteria occupy specific niches within the coral host, such as the surface mucus layer and tissue interstices, where they contribute to key processes including nutrient cycling, metabolic exchange, and immune modulation, thereby directly influencing the coral’s ability to adapt to environmental change. Taxonomically, these communities are highly diverse, often dominated by groups such as Proteobacteria, Bacteroidetes, and Actinobacteria, yet their composition exhibits marked host-specificity and environmental plasticity. This means the dominant bacterial taxa at the genus or species level can shift substantially across different coral species, geographic locations, or under varying environmental stressors, reflecting the community’s capacity to respond to and potentially mitigate external pressures. Besides, these bacteria support coral health through mechanisms such as nitrogen fixation, organic phosphate solubilization, and vitamin synthesis, while also aiding in pathogen resistance via antimicrobial production or competitive exclusion. Therefore, integrating knowledge of the taxonomic structure, ecological roles, and physiological pathways of coral-associated bacteria is essential for unraveling the mechanisms of coral-bacterial symbiosis, assessing the adaptive potential of corals under climate change, and informing microbiome-based strategies for reef conservation.

Coral symbiotic bacteria confer advantages to their hosts through multiple mechanisms, facilitating coral survival and reproduction in complex marine environments. These bacteria contribute to coral health through essential physiological mechanisms, including nitrogen fixation (Pogoreutz et al., 2017), organic phosphate solubilization (Godinot et al., 2011), and vitamin synthesis (Maire et al., 2023), while also enhancing pathogen resistance via antimicrobial production or competitive exclusion (Raina et al., 2016). It is worthwhile mentioning that beneficial microorganisms for corals (BMC) isolated from coral hosts have demonstrated significant roles in helping corals cope with environmental stressors (Peixoto et al., 2017). The judicious application of BMC can enhance coral nutrient uptake and growth (Li et al., 2023b), mitigate environmental stress (Rosado et al., 2023) and toxic compound effects, prevent pathogen invasion (Gochfeld and Aeby, 2008), support early life-stage development, and strengthen coral resilience to climate change.

Notably, the microbial community composition on coral surfaces is not static; rather, it is largely regulated by the coral’s environmental conditions (Ziegler et al., 2019). Different coral species, and even the same species under varying environmental conditions, may exhibit differences in their bacterial community composition (Roder et al., 2015). Therefore, gaining a thorough understanding of the bacterial community composition across different corals and identifying general patterns in microbial distribution are essential prerequisites for elucidating the symbiotic relationship between corals and microorganisms (Mohamed et al., 2023).

Currently, despite widespread recognition of the importance of coral-associated bacteria, determination and detection of bacteria with traditional biochemical assays are inherently time-consuming and laborious tasks (Babaie et al., 2021). Ideally, it takes at least a few hours until the results are available, but, depending on the type of bacteria, it normally takes much longer (Lorenz et al., 2017). This has resulted in a lack of in-depth understanding regarding the specific interaction mechanisms between corals and bacteria, as well as the dynamic patterns of microbial community changes. Further research in this area is urgently needed (Zhu et al., 2023), the field of coral microbiome research has established a methodology system centered on multi-omics integration. Through the combination of metagenomics, metatranscriptomics, and metabolomics, coupled with the rise of high-throughput sequencing technologies, most laboratories are now capable of comprehensively sequencing cellular RNA and DNA (Zhou et al., 2018). This integrated approach facilitates not only in-depth analysis of microbial community composition but also elucidates their functional activity and metabolic characteristics, opening new avenues for understanding bacterial gene expression and regulation. This integrated research strategy allows the academic community to systematically elucidate the functional roles of microorganisms within coral symbiotic systems at the molecular level (Gong et al., 2023; Jaafar et al., 2023; Wong et al., 2024).

Daya Bay (22°31′-22°50′N, 114°29′-114°49′E) is one of China’s few coastal bays hosting reef-building corals. Though climate constraints prevent full reef development, it supports multiple high-latitude coral communities. These communities comprise relatively resilient species such as stony coral species (Li et al., 2008). This study utilized high-throughput sequencing of the 16S rRNA gene V4 region and subsequent bioinformatics analyses, to reveal compositional differences and diversity characteristics within bacterial communities in four species of stony corals *Favites abdita* (*Fa*), *Favia speciosa* (*Fs*), *Montipora digitata* (*Md*) and *Porites solida* (*Ps*) from Daya Bay, thereby predicting their core metabolic functions. This study not only enriches baseline data on microbial diversity in South China Sea corals but also provides crucial microbiological evidence for coral reef health assessment and ecological conservation.

## 2 Materials and methods

### 2.1 Sample collection, preparation and identification

**Figure 1:**
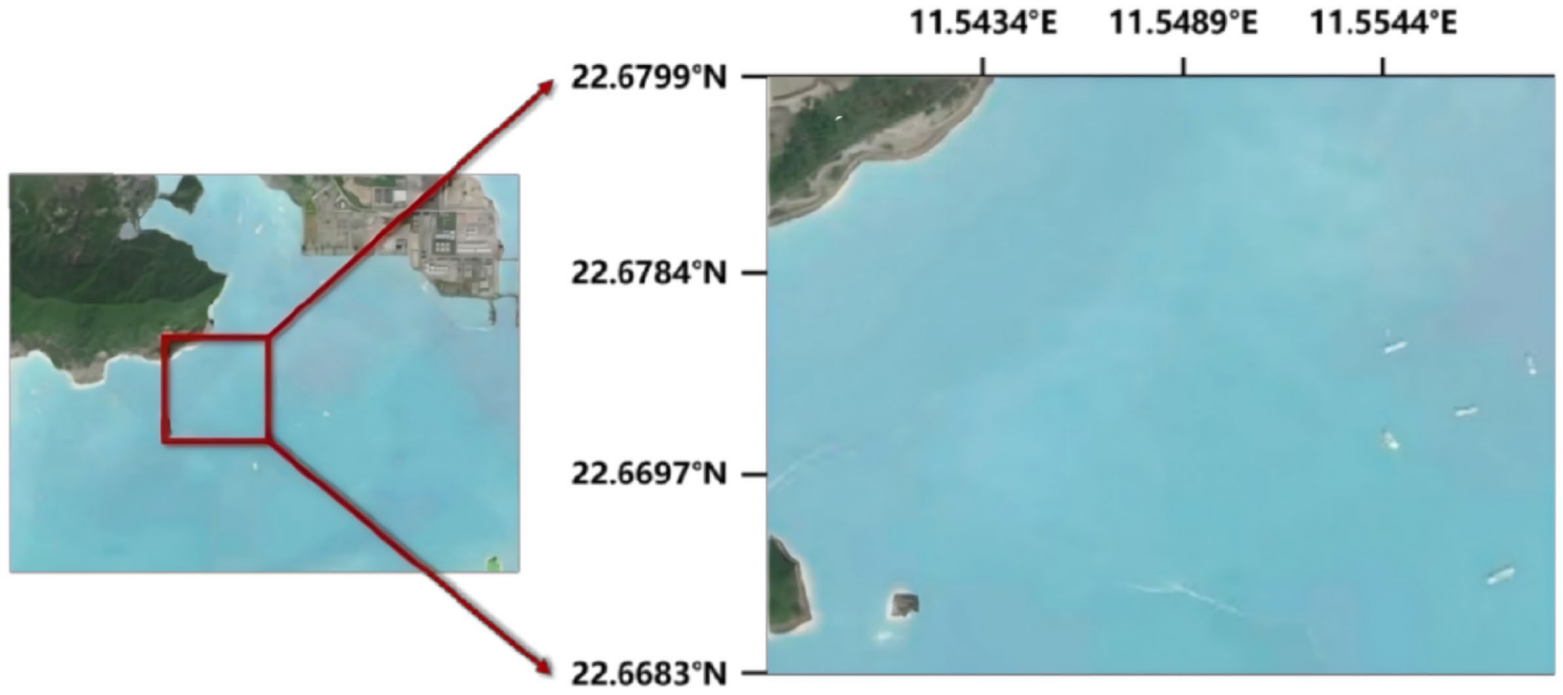
Map of the Daya Bay in the South China Sea and the location of the coral sampling site. The map shows the sampling point coordinates as 22°40′37″ North Latitude, 114°32′59″ East Longitude.

Four species of stony corals were collected from the offshore area of Daya Bay in the South China Sea (22°40′37″N, 114°32′59″E) (Figure 1). Prior to sampling, precise coordinates were marked using a GPS, confirming a water depth of 11 meters in the sampling area. While ensuring coral integrity, target species were first identified and marked *in situ* as *F. abdita*, *F. speciosa*, *M. digitata*, and *P. solida* (abbreviated as *Fa*, *Fs*, *Md*, and *Ps*, respectively). Samples were then collected using specialized tools to avoid damaging the coral parent structure. They were then transferred to sterile plastic bags and documented with sampling time, location, and depth. Samples were transported to the laboratory on ice and promptly transferred to a − 20 °C freezer upon arrival for cryopreservation (Zhang et al., 2015), thereby preserving the original structure and composition of the microbial communities. Samples from the *Fa*, *Fs*, *Md*, and *Ps* groups were assigned to subgroups: *Fa*1–*Fa*3, *Fs*1–*Fs*3, *Md*1–*Md*3, and *Ps*1–*Ps*4. The sample collection procedure has been approved by the Animal Ethics Committee of South China Agricultural University, Guangzhou, China (Approval Number: SYXK-2019-0136).

**Figure 2:**
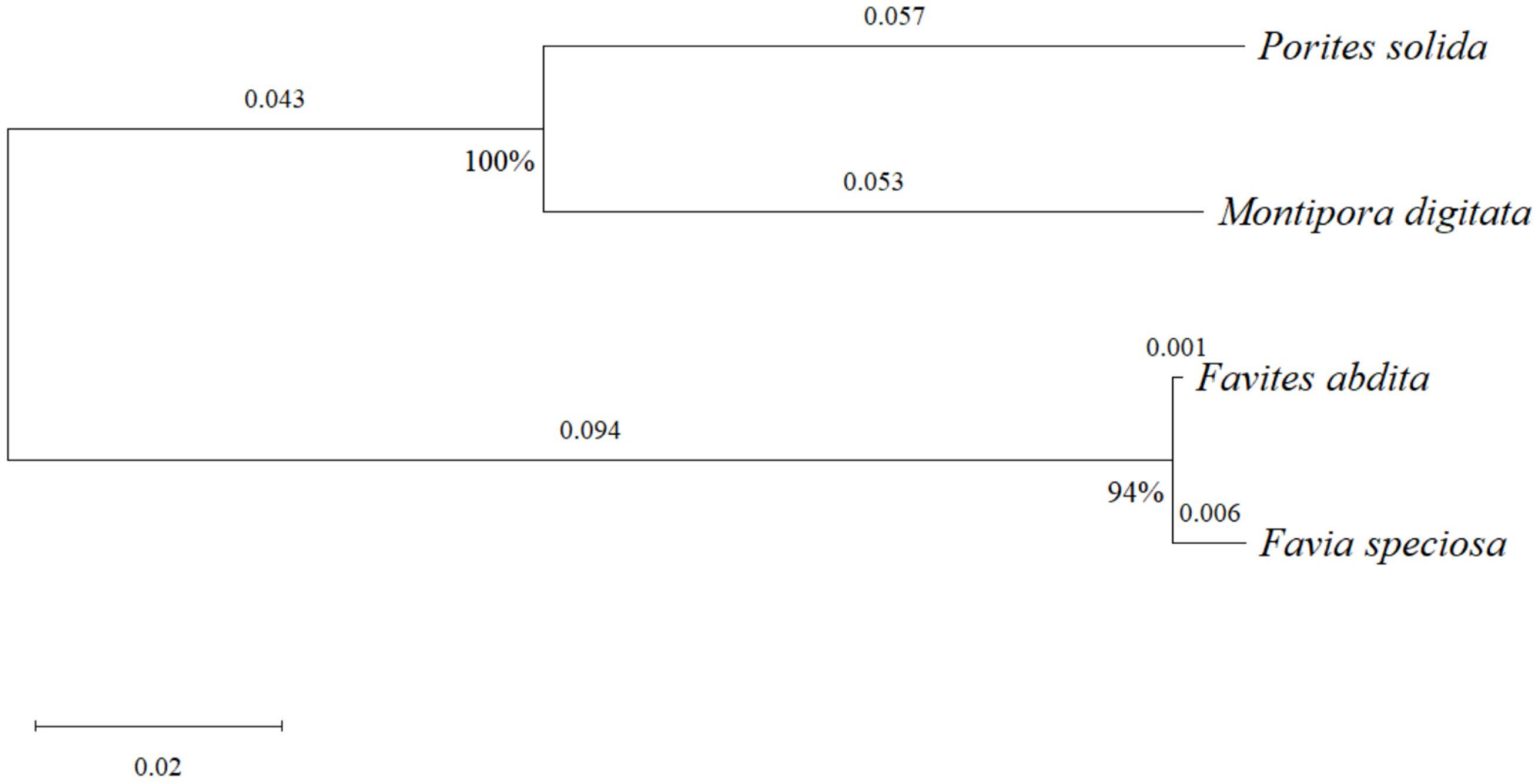
Neighbor-joining phylogenetic tree based on cytochrome c oxidase subunit I (COI) gene sequences from the four South China Sea coral species. The tree was constructed using the maximum composite likelihood method in MEGA 11.0, with nodal support based on 1,000 bootstrap replicates. Bootstrap support values are shown at nodes, illustrating the evolutionary relationships among the coral species.

To investigate the evolutionary relationships and genetic diversity of the coral specimens, the cytochrome c oxidase subunit I (COI) gene—a widely used and reliable molecular marker for species identification (Dong et al., 2024)—was analyzed. A phylogenetic tree was subsequently constructed from COI sequences retrieved from the National Center for Biotechnology Information (NCBI) database to resolve the phylogenetic relationships among the four species (Figure 2).

### 2.2 DNA extraction and high-throughput sequencing

Each intact small coral sample was pretreated in a laminar flow hood. The sample surfaces were first rinsed with 75% ethanol solution, followed by three rinses with sterile seawater to remove loosely attached microorganisms and mucus (Liu et al., 2021; Silva et al., 2023). Each coral sample was crushed in a sterile mortar and pestle, and approximately 1.0 milliliter of homogenate was collected. Genomic DNA extraction was performed strictly according to the operating instructions of the AxyGen AxyPrep DNA Gel Recovery Kit. Following extraction, the integrity of the genomic DNA was verified by 1% agarose gel electrophoresis to ensure suitability for subsequent PCR amplification experiments.

Using extracted genomic DNA as a template, universal primers were designed for PCR amplification targeting the V4 variable region of the bacterial 16S rRNA gene (Liu et al., 2018). The forward primer is 515F (5′-GTGCCAGCMGCCGCGGTAA-3′) and the reverse primer is 907R (5′-CCGTCAATTCMTTTRAGTTT-3′). The PCR reaction system utilized TransGen Company’s AP221-02 model TransStart Fastpfu DNA Polymerase enzyme. After setting up the program, the reaction was carried out on a PCR amplification instrument (Magoč and Salzberg, 2011). The PCR thermal cycling program is set as follows: Initial denaturation at 94 °C for 5 min, followed by 31 cycles. Each cycle consists of denaturation at 94 °C for 30 s, annealing at 53 °C for 30 s, and extension at 72 °C for 45 s. Finally, extend at 72 °C for 10 min. After the reaction, store the product at 4 °C.

After pooling PCR products from the same sample, amplification efficiency was assessed via 2% agarose gel electrophoresis. Gel digestion and recovery were performed using the AxyGen AxyPrep DNA Gel Recovery Kit to obtain purified PCR products for preliminary quantification. Subsequently, precise quantification was achieved using the Promega QuantiFluor™-ST Blue Fluorescent Quantification System (Soldevila-Boixader et al., 2025). Based on quantitative results, PCR products from different samples were mixed proportionally. Sequencing work was commissioned to Shanghai Meiji Biotechnology Co., Ltd.

### 2.3 Sequence analysis

Raw paired-end sequencing reads underwent quality control using fastp and were assembled with FLASH (Magoč and Salzberg, 2011), and then use UPARSE v7.1 software to assign operational taxonomic units (OTUs) to the assembled sequences based on 97% similarity. Taxonomic annotation of the OTUs was performed by aligning sequences against the Silva 16S rRNA gene database (v138) with the RDP classifier at a 70% confidence threshold. Finally, community composition was statistically analyzed for each sample at different taxonomic levels.

### 2.4 Diversity analysis

Analyses of diversity metrics included rarefaction curves, α-diversity (Chao1, Shannon, ACE, Simpson, coverage), and β-diversity (Principal Coordinate Analysis, PCoA) (Dong et al., 2024). The α-diversity indices, including Chao1 and Shannon, were calculated using the mothur software. Significant differences in α-diversity between groups were evaluated using Wilcoxon rank-sum tests. PCoA based on the Bray-Curtis distance algorithm was employed to assess microbial community structure similarity among samples. Combined with PERMANOVA nonparametric testing, this approach evaluated whether microbial community structure differences between sample groups were statistically significant.

### 2.5 Function prediction analysis

Function prediction analysis was primarily performed using the Tax4Fun software package (Sun et al., 2024). This involved extracting 16S rRNA gene sequences from prokaryotic genomes in the KEGG database and aligning them against the SILVA SSU RefNR database using the BLASTN algorithm (BLAST bit score > 1,500) to establish a corresponding matrix. Functional annotations from the KEGG database for prokaryotic whole genomes, annotated with both UProC and PAUDA, were mapped to the SILVA database. This mapping enables functional annotation of the SILVA database, which subsequently serves as a reference for aligning query sequences to obtain functional information.

### 2.6 Nucleotide sequence accession number

The 16S rRNA gene sequences corresponding to all coral samples have been submitted to the Sequence Read Archive of the National Center for Biotechnology Information and are accessible under the accession number SUB15798809.

## 3 Results

### 3.1 Bacterial community composition

**Figure 3:**
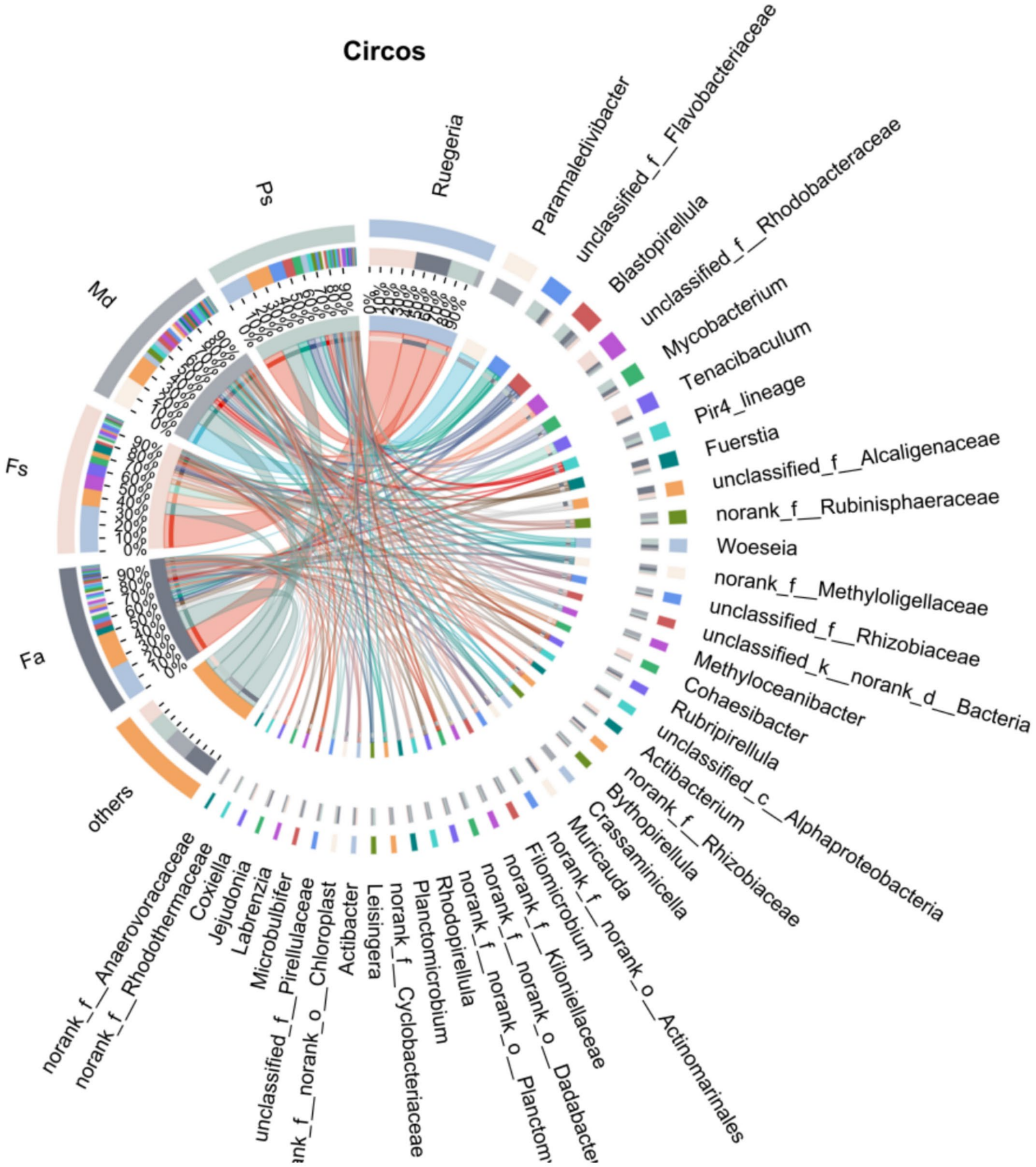
Circos plot of major bacterial phyla’s relative abundance and associations. *Fa*, *Favites abdita*; *Fs*, *Favia speciosa*; *Md*, *Montipora digitata*; *Ps*, *Porites solida*.

This research analyzed the bacterial communities in the samples using a culture-independent technique. Bacterial taxonomic units belonging to 23 phyla and 250 genera were identified from all samples. Circos plots (Figure 3) visually display the relative abundance of major bacterial phyla across different sample groups and their interrelationships, as well as the similarities and differences in the composition of bacterial communities in different samples. The four biological groups exhibit high overlap in their genus-level distribution.

**Figure 4:**
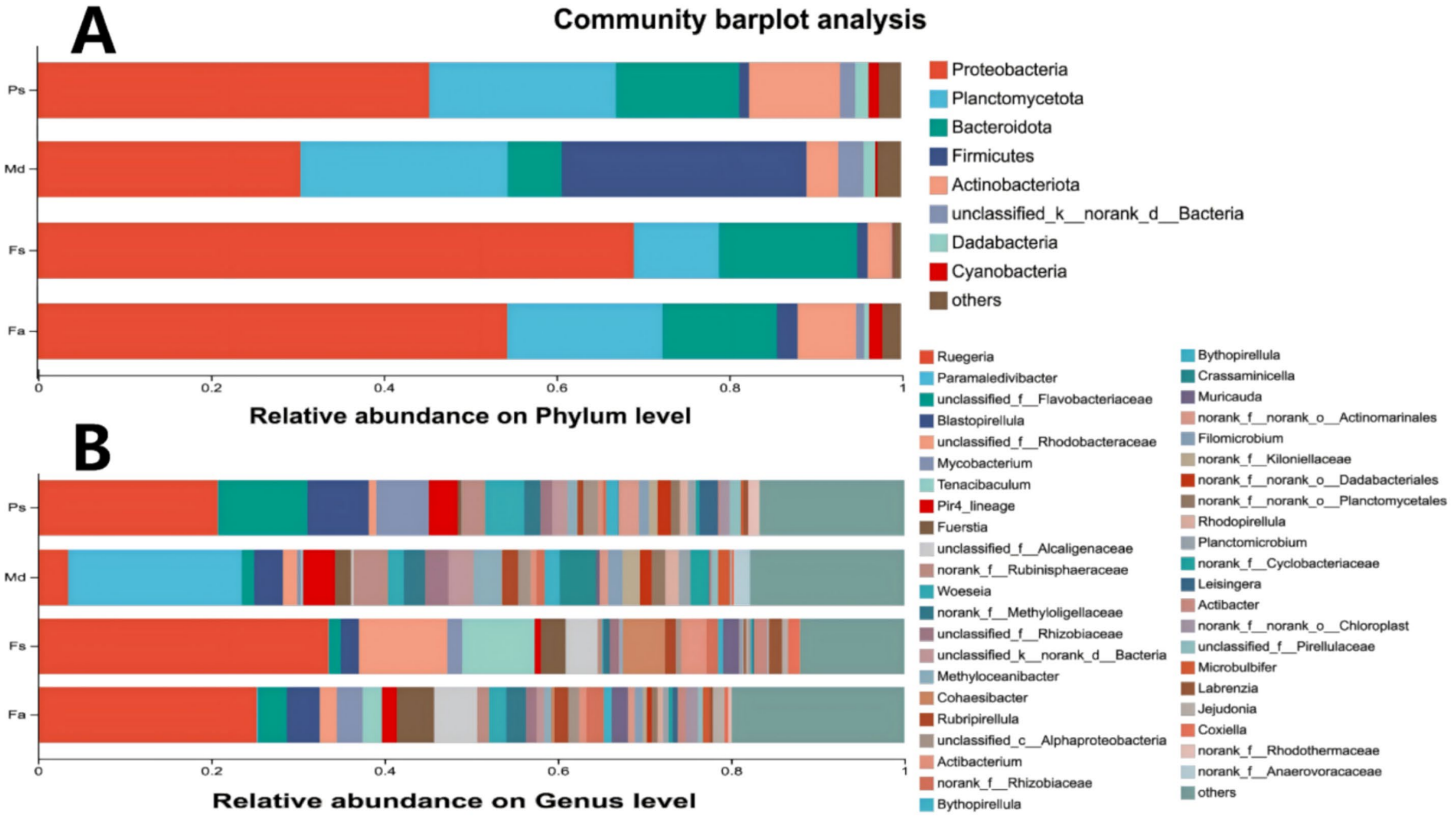
Phylum-level abundance **(A)**, *FA*, *FS*, *PS* dominated by Proteobacteria, *MD* by Firmicutes. Genus-level abundance **(B)**, *FS* dominated by *Ruegeria*, *MD* with no single dominant genus. *FA*, *Favites abdita*; *FS*, *Favia speciosa*; *MD*, *Montipora digitata*; *PS*, *Porites solida*.

Significant differences in dominant bacterial phyla were observed among sample groups at the phylum level (Figure 4A; Supplementary Table S1). Corals *Fa*, *Fs*, and *Ps* exhibited absolute dominance by the Proteobacteria phylum, followed by accompanying groups such as the Proteobacteria and Bacteroidetes phyla. In contrast, *Md* samples showed dominance by the Firmicutes phylum.

At the genus level, community structure exhibited greater complexity (Figure 4B; Supplementary Table S2). Each sample group displayed unique dominant genus combinations and community structural characteristics. Although the corals *Fa* and *Ps* were both dominated by *Proteobacteria*, their bacterial communities at the genus level were sharply divergent. Coral *Fs* featured the hyperdominant genus *Ruegeria* at the genus level. In contrast, the *Md* coral displayed high bacterial diversity, characterized by an even distribution of multiple genera and the absence of a dominant taxon.

### 3.2 Comparison of community structure diversity

**Figure 5:**
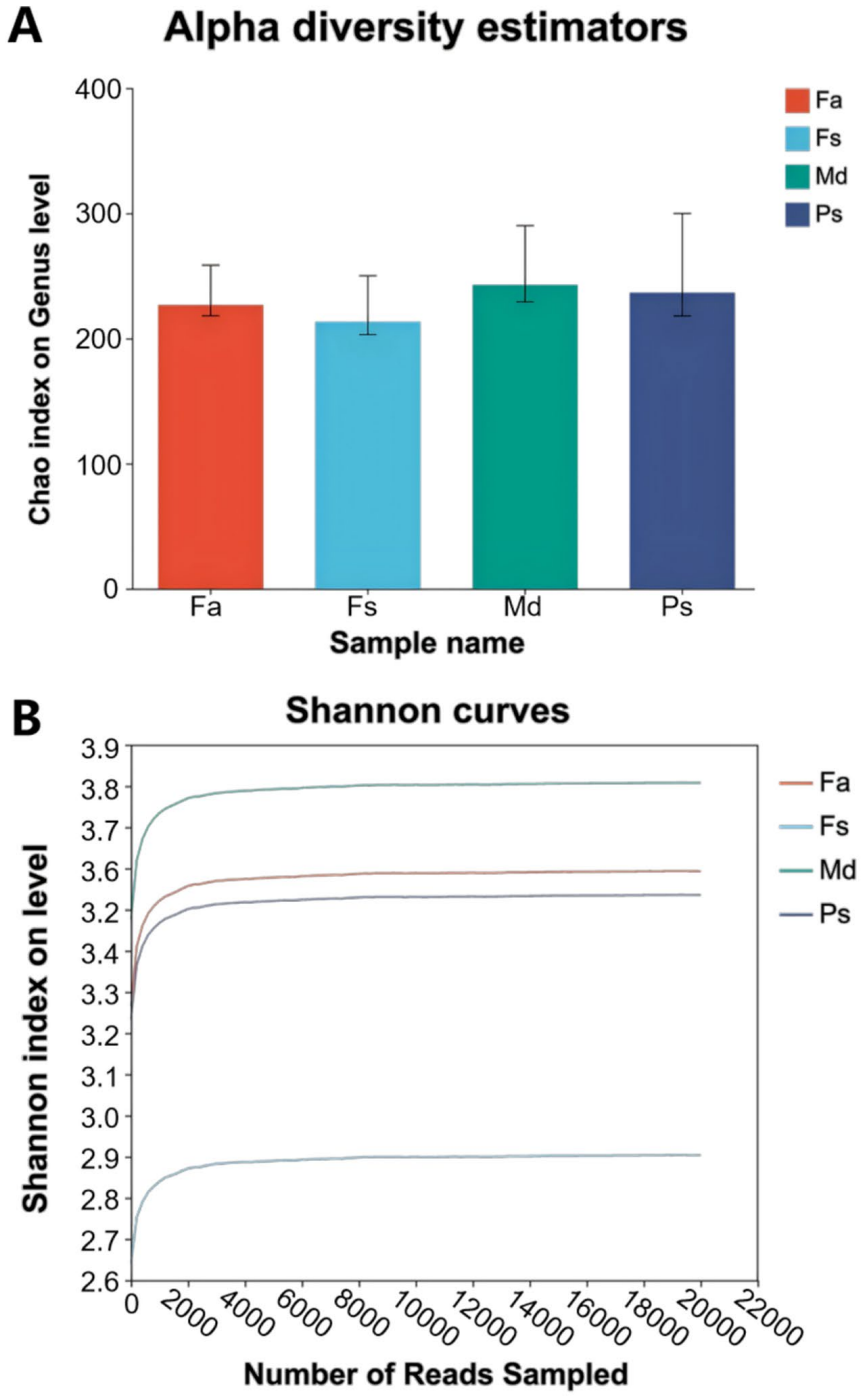
Alpha diversity bar chart **(A)**, which has key result *Md* has the highest diversity and *Fs* the lowest. Rarefaction curves **(B)**. *Fa*, *Favites abdita*; *Fs*, *Favia speciosa*; *Md*, *Montipora digitata*; *Ps*, *Porites solida*.

Community richness and diversity were assessed using multiple indices. The bar chart of the alpha diversity index (Figure 5A) illustrates differences in community richness and diversity among sample groups, with coral *Md* exhibiting the highest species abundance and coral *Fs* the lowest. To assess the adequacy of sequencing depth, rarefaction curves were plotted (Figure 5B). All curves flattened at their ends, with coverage exceeding 99% across all corals (Table 1), indicating that the current sequencing data sufficiently cover the vast majority of bacterial species in the samples. Shannon, Simpson, ace, and Chao1 indices (Table 1) demonstrated consistent bacterial abundance and diversity across these 13 groups.

**Table 1:** Diversity indices of the Shannon, Simpson, Ace, Chao1 and coverage indices.

Samples	Shannon	Simpson	Ace	Chao1	Coverage
*Fa*1	3.94	0.04	241.11	242.00	99.86%
*Fa*2	3.32	0.12	216.07	213.32	99.89%
*Fa*3	3.52	0.10	226.85	224.65	99.91%
*Fs*1	2.86	0.13	209.67	218.07	99.86%
*Fs*2	2.88	0.21	212.64	209.54	99.87%
*Fs*3	2.97	0.15	214.27	212.13	99.90%
*Md*1	3.62	0.09	248.80	243.24	99.86%
*Md*2	3.89	0.04	242.50	254.43	99.84%
*Md*3	3.92	0.05	227.80	230.40	99.89%
*Ps*1	3.55	0.08	223.91	228.00	99.88%
*Ps*2	3.40	0.09	213.17	211.00	99.89%
*Ps*3	3.53	0.07	229.25	244.33	99.84%
*Ps*4	3.66	0.05	248.91	262.67	99.80%

The coral species analyzed included *Favites abdita* (*Fa*), *Favia speciosa* (*Fs*), *Montipora digitata* (*Md*), *Porites solida* (*Ps*). Shannon and Simpson indices measure species diversity and evenness, while Richness, Chao1, and Ace indices estimate species richness, Coverage reflects the proportion of this data that falls within this group.

**Figure 6:**
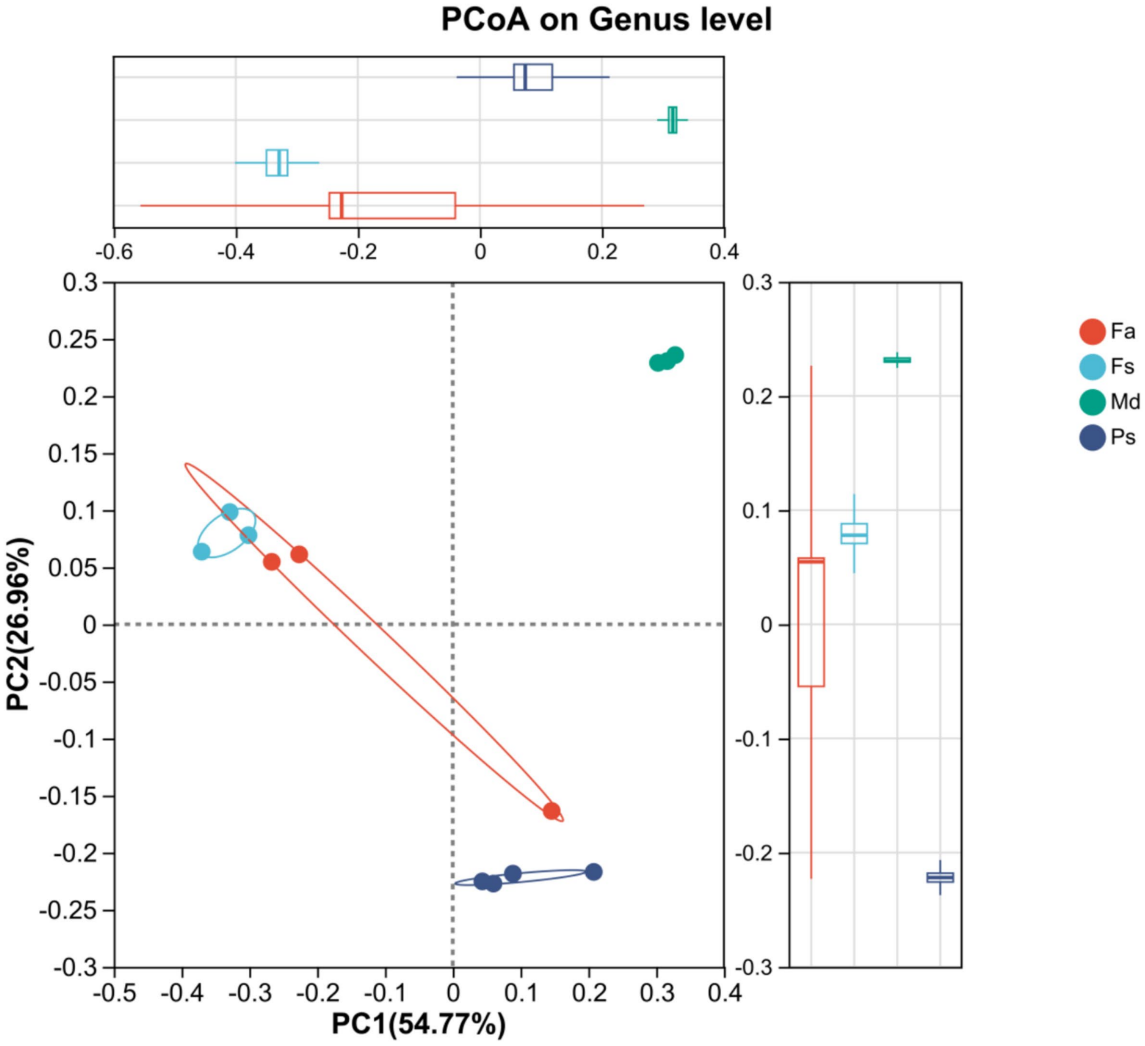
PCoA plot. *Md* separated from others, *Fa* overlaps with *Fs*. *Fa*, *Favites abdita*; *Fs*, *Favia speciosa*; *Md*, *Montipora digitata*; *Ps*, *Porites solida*.

Principal coordinate analysis (PCoA) based on Bray-Curtis distance was employed to assess differences in community structure (beta diversity) among sample groups. The PCoA scatterplot (Figure 6) revealed that principal coordinate 1 (PC1) and principal coordinate 2 (PC2) explained 54.77 and 26.96% of the total variation in community structure, respectively, with a cumulative explanation of 81.73%. The samples exhibited distinct spatial separation and clustering patterns in the plot. Coral *Md* was most distant from other corals, while corals *Fa* and *Fs* showed significant overlap. Although coral *Ps* did not overlap with other corals, it was not markedly different from corals *Fa* and *Fs*.

**Figure 7:**
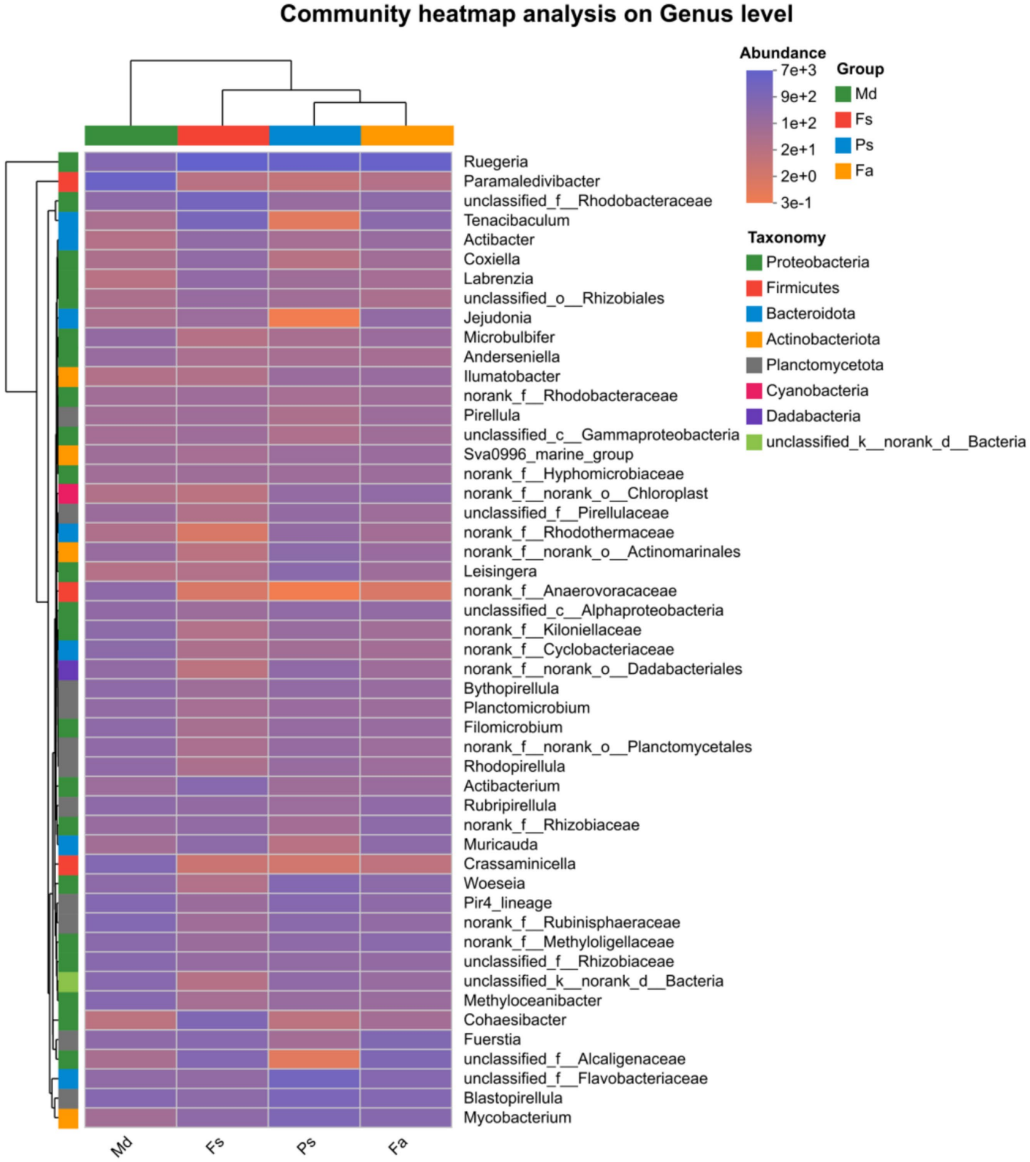
Genus-level heatmap, which displays abundance-based clustering. *Fa*, *Favites abdita*; *Fs*, *Favia speciosa*; *Md*, *Montipora digitata*; *Ps*, *Porites solida*.

To further visualize the similarity in community composition at the genus level across different samples, a community heatmap was generated (Figure 7). This map reflects the relative abundance of major bacterial genera through a color gradient, illustrating the clustering relationships between samples and genera based on their abundance levels. The bacterial abundance profiles of the *Fa* and *Fs* groups were broadly similar, except for a lower relative abundance of the family *Rhodothermaceae* in *Fs*. In comparison, overall bacterial abundance was reduced in the *Ps* group, while the *Md* group presented a distinct profile dominated by the genus *Paramaledivibacter*. Conversely, the genus *Ruegeria* was consistently present and relatively abundant in the remaining coral groups.

### 3.3 Prediction of community functional potential

**Figure 8:**
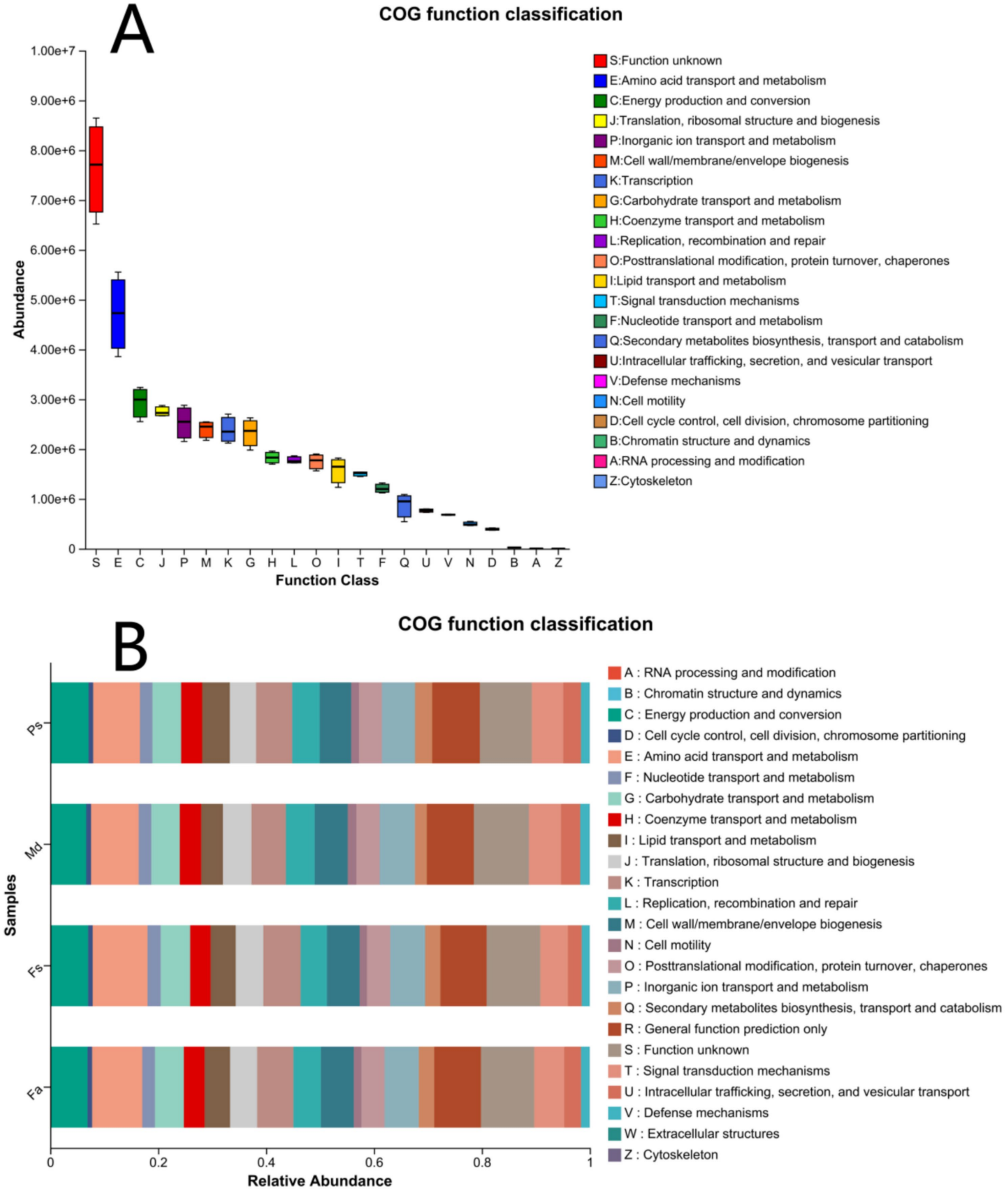
COG function classification plot of all sample **(A)**. COG function classification plot of four groups (*Fa*, *Fs*, *Md*, *Ps*) of coral microorganisms **(B)**. *Fa*, *Favites abdita*; *Fs*, *Favia speciosa*; *Md*, *Montipora digitata*; *Ps*, *Porites solida*.

Analysis of Clusters of Orthologous Genes (COG) functional categories revealed that, excluding the Function unknown (S) category, the functional groups with the highest abundance and widest distribution were those associated with core functions: Amino acid transport and metabolism (E), Energy production and conversion (C), and Translation, ribosomal structure and biogenesis (J) exhibited the highest abundance and broadest distribution (Figure 8A). Moreover, the relative proportions of these four functional groups were highly consistent across the four coral species *Fa*, *Fs*, *Md*, and *Ps* (Figure 8B). Thus, despite differences in bacterial community structure, core metabolic functions remain highly conserved—an integral feature of the coral holobiont.

**Figure 9:**
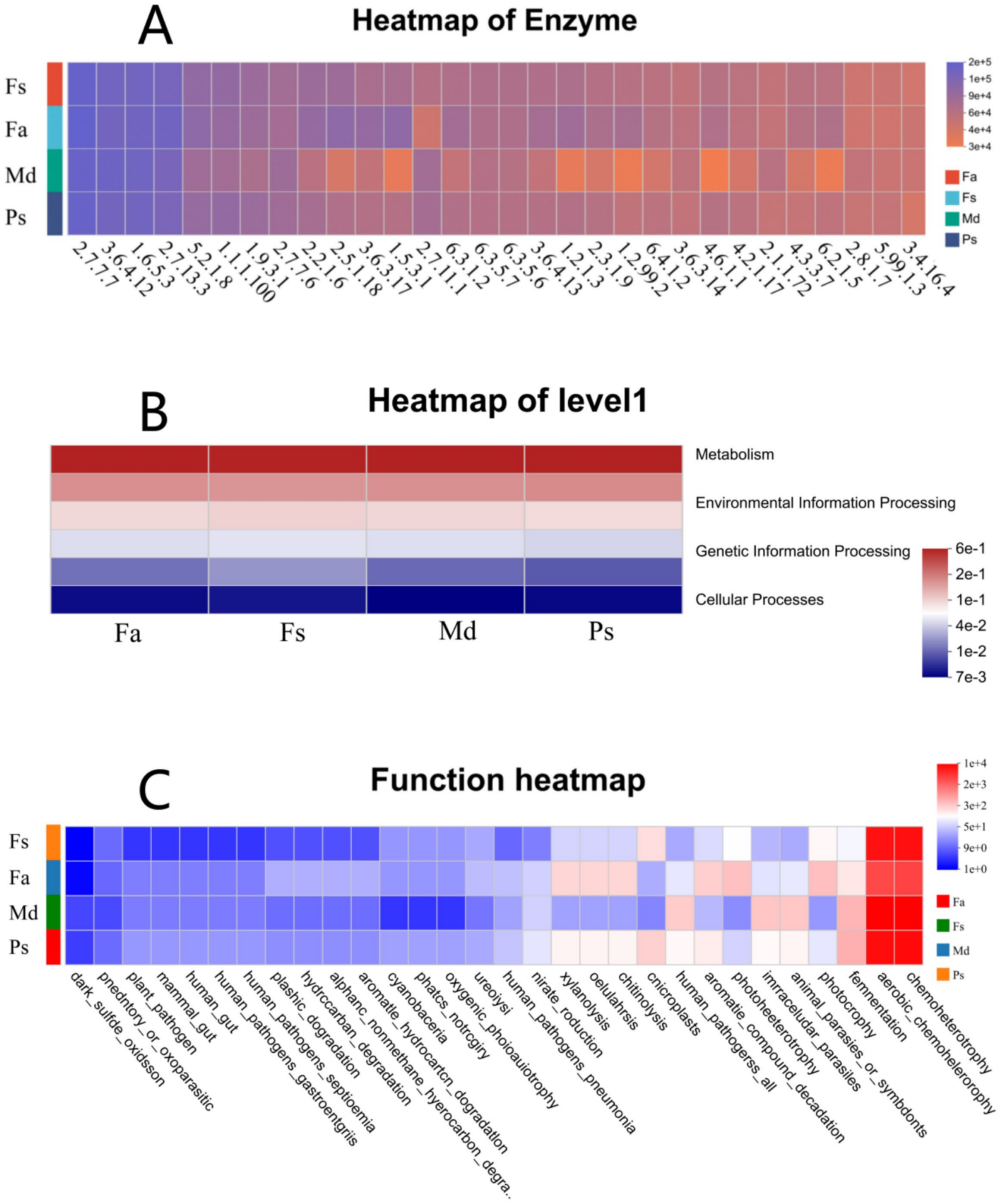
Enzyme abundance heatmap highlights inter-coral differences via color gradients **(A)**. Functional category plot shows metabolic functions most abundant **(B)**. Functional phenotypic heatmap of bacterial communities associated with four stony coral species **(C)**. *Fa*, *Favites abdita*; *Fs*, *Favia speciosa*; *Md*, *Montipora digitata*; *Ps*, *Porites solida*.

At the enzyme functional level, the distribution of enzyme abundances across the four sample groups were broadly similar but exhibited distinct variations (Figure 9A). Corals *Fa* and *Ps* exhibited the most similar enzyme abundance profiles, with relatively uniform distributions. Coral *Fs* showed a pronounced dominance of enzyme 2.7.11.1, while the remaining enzyme classes were present at relatively low and evenly distributed abundances. Coral *Md* exhibited the richest enzymatic functional diversity, with multiple enzymes maintaining high abundances, showing significant differences from the other three corals.

At the functional category level, heatmap analysis revealed that the functional compositions of the four coral samples exhibited high consistency at the highest functional hierarchy (KEGG Level 1) (Figure 9B). Among these, the relative abundance of Metabolism was significantly higher than other categories, forming the core functional module of the bacterial community. Environmental Information Processing and Genetic Information Processing functions followed in importance, while functions related to Cellular Processes, Human Diseases, and Organismal Systems accounted for an extremely low proportion. These findings corroborate the conserved core functions identified in the COG analysis, indicating that despite differences in microbial community structures among coral species, they exhibit high consistency in core functions.

Further heatmap analysis based on specific metabolic phenotypes revealed significant divergence among different coral samples across multiple functional potentials (Figure 9C). Specifically, coral *Md* exhibited strong potential in xylanolysis, cellulolysis, chitinolysis, aromatic compound degradation, photoheterotrophy, and fermentation, while *Fs* demonstrated outstanding hydrocarbon degradation. *Fa* and *Ps* showed closer similarities across most metabolic phenotypes. These results indicate that despite a highly conserved overall functional spectrum, different coral symbiotic bacterial communities exhibit adaptive differentiation in specific ecological functions such as nitrogen and sulfur cycling, carbon source utilization, and energy metabolism. This differentiation may be related to differences in their microenvironments and host-specific adaptation strategies.

## 4 Discussion

The interaction between corals and microorganisms is one of the fundamental dynamics on coral reefs (Wegley et al., 2007). Corals are increasingly facing changing environmental conditions at both regional and global scales (Bozec et al., 2025). To understand how these changes will affect the coral symbiotic system as a whole, it is necessary to understand the role of microorganisms and their responses to these changes (Li et al., 2023a). All current evidence indicates that corals and their microbial symbionts exist in a delicate state of equilibrium.

Analysis of samples following high-throughput sequencing revealed that this study identified 368 bacterial species belonging to 250 genera across 23 phyla from four coral groups. This finding demonstrates the remarkable diversity of bacterial communities within coral symbiotic microenvironments, establishing a taxonomic foundation for subsequent investigations into the functional maintenance mechanisms of coral-microbe symbiotic systems. Among these, Proteobacteria accounted for the largest proportion, comprising 49.52% of the total sequences, consistent with the distribution patterns of coral microorganisms in most regions (Lan et al., 2025; Rabbani et al., 2025). Second is the Planctomycetota phylum, accounting for 18.61% of total sequences. The bacterial group distributions at the genus level showed high overlap among the four corals *Fa*, *Fs*, *Md*, *Ps*, suggesting the potential presence of shared core bacterial groups. These core groups likely serve as key components that perform fundamental ecological functions within the coral microenvironment, such as nutrient cycling and maintaining host microenvironmental homeostasis, reflecting the functional conservation of coral-associated bacterial communities (Jessen et al., 2013). These taxa can be regarded as specialized adaptive components of the community tailored to specific habitat conditions, directly reflecting the microbial community’s response and differentiation characteristics in response to different microhabitats (Cai et al., 2018; Gong et al., 2020; Pootakham et al., 2021).

The absolute dominant phyla in corals *Fa*, *Fs*, and *Ps* were all Proteobacteria, accompanied by distributions of Planctomycetes, Bacteroidetes, and other groups. This finding is consistent with the conclusions of most studies on coral symbiotic microorganisms (Quek et al., 2023). The dominant phylum in coral *Md* is Firmicutes (Figure 4). These Gram-positive bacteria are predominantly spore-forming, capable of tolerating extreme environments such as low oxygen levels and high organic matter content, and exhibit strong anaerobic metabolic capabilities (Villegas-Plazas et al., 2019). Interestingly, the *Md* group and other coral groups were collected from the same location, so such pronounced differences in their Symbiodinium would not be expected. The reason may lie in the distinct symbiotic dinoflagellates associated with *Md* group corals compared to other corals, leading to the formation of different Symbiodiniaceae Phycospheres (Gantt et al., 2024). Due to the vertical transmission of *Symbiodinium* (Damjanovic et al., 2020), the *Symbiodinium* species within the *Md* group undergo minimal change. Consequently, these differences accumulate over time, resulting in microbial communities that are distinctly different from those observed in other groups.

Within the horizontal structure of the Fs group, the genus *Ruegeria* exhibits extreme dominance (Figure 4). This is the same as the results reported previously (Sogin et al., 2017). Previous studies have confirmed that some species within this genus can participate in biofilm formation, produce quorum-sensing signaling molecules, or generate antimicrobial substances (Zhang et al., 2017; Baek et al., 2020). It is hypothesized that within coral *Fs*, *Ruegeria* maintains microenvironmental equilibrium by suppressing harmful bacteria and stabilizing community structure (Kitamura et al., 2021). Coral *Md* exhibits high diversity at the genus level, with relatively even distribution of abundance across genera and no single dominant genus (Figure 4). This structure is typically associated with highly heterogeneous habitat resources, allowing different taxa to occupy distinct ecological niches and engage in synergistic metabolism, thereby enhancing the community’s resilience to disturbances (Heino et al., 2013; Mcleish et al., 2024). Although corals *Fa* and *Ps* are both dominated by the Proteobacteria phylum, their dominant genera exhibit species differentiation. The observed variations in microbial communities may be due to the corals being distinct species, which lead to varying competitive advantages among different Proteobacteria genera, ultimately forming unique genus-level compositions.

A systematic assessment of community richness and diversity not only revealed significant differences among the four corals but also validated the reliability of the research data, providing a solid foundation for subsequent analyses of community structure and function. Alpha diversity characteristics showed that coral *Md* exhibited the highest species abundance, while coral *Fs* displayed the lowest species abundance (Figure 5). This result closely aligns with the community composition patterns observed in both groups. The high species abundance in coral *Md* aligns with its structural characteristics of high diversity at the genus level and the absence of a single dominant genus. This high diversity ensures the stability of its community functions, a feature that further supports the hypothesis that coral *Md*′s habitat exhibits high heterogeneity (Janousek et al., 2007; Dong et al., 2024). The low species richness in coral *Fs* may be associated with the extreme dominance of the genus *Ruegeria* (Figure 5). Dominant genera can suppress the growth of other species through resource competition, such as competing for carbon and nitrogen sources, ultimately forming a community structure dominated by a single dominant species. Although such structures exhibit lower diversity, they maintain community stability through the efficient functions of dominant species (e.g., *Ruegeria*’s defense capabilities), representing a common strategy for microbial communities to adapt to specific habitats (Poulin et al., 2006).

The rarefaction curves for all species groups level off at their ends, indicating that the current data volume is sufficient to capture the vast majority of bacterial species in the sample. No further increase in data volume is required to ensure the completeness of species detection (Fernando et al., 2015).

Meanwhile, the coverage rate of samples across all groups exceeded 99%, further confirming the comprehensiveness of the data (Table 1). Regarding the numerical characteristics of alpha diversity indices (Shannon, Simpson, Ace, Chao1) (Chen et al., 2024), the trends across groups were consistent. For instance, coral *Md* generally exhibited higher Shannon indices than coral *Fs*, while Ace and Chao1 indices also showed higher values in coral *Md*. This quantitatively corroborates the diversity differences among sample groups, ensuring the reliability of subsequent analysis results. Interestingly, the Chao1 index indicates that coral *Ps* exhibits slightly higher diversity than coral *Fa*, yet the Shannon index reveals the opposite relationship between the two corals (Figure 5). This discrepancy may arise because the Chao1 index reflects species richness, whereas the Shannon index accounts for both species richness and species evenness. Coral *Fa* contains more rare species, resulting in slightly higher species richness than coral *Ps*. However, the relatively small number of rare species leads to lower species evenness. In contrast, coral *Ps* exhibits slightly lower species richness but significantly more even species distribution compared to coral *Fa*. Consequently, while coral *Ps* has a lower Chao 1 index than coral *Fa*, its Shannon index is higher.

Intergroup differences in community structure further reveal the differentiation patterns among samples. From the perspective of spatial distribution characteristics, coral *Md* exhibits the most pronounced differentiation from the other three corals. This aligns closely with coral *Md*’s unique composition: dominance at the phylum level by the phylum Actinomycetes and high diversity without dominant genera at the genus level. This indicates that its community structure fundamentally differs from the other three groups. Coral *Fa* and coral *Fs* exhibit high similarity in community structure and show significant spatial overlap (Figure 6). This phenomenon may stem from the closer physicochemical conditions, such as oxygen concentration and host type in their respective habitats (Van De Water et al., 2022), resulting in smaller differences in bacterial community survival advantages. Although coral *Ps* does not share overlapping regions with other corals, its close phylogenetic relationship with coral *Fa* and *Fs* indicates that despite its unique community structure, it retains numerous similar characteristics with coral *Fa* and *Fs*.

From the distribution of COG functional categories (Figure 8), aside from functions of unknown significance, the core clusters of amino acid transport and metabolism, energy production and conversion, and translation and ribosomal structure/biogenesis exhibit the highest abundance and broadest distribution, reflecting the fundamental life activities of bacteria. These three vital activities are crucial for coral symbiotic microorganisms and play a significant role in maintaining their vital functions. Notably, the four corals *Fa*, *Fs*, *Md*, *Ps* exhibited highly consistent relative proportions across major COG functional categories. This shared functional composition indicates that despite divergent bacterial community structures among the four corals, the core functional requirements supporting basic microbial survival and metabolism remained largely unchanged. This demonstrates the functional conservatism of coral-associated microbial communities (Zhu et al., 2023). This conservatism may underpin microbial adaptation to coral habitats and the maintenance of stable mutualistic interactions with hosts, while also reflecting the role of functional redundancy in sustaining community stability (Abdelghany et al., 2025).

At the level of enzyme abundance distribution (Figure 9), the four corals exhibited both overall similarity and distinct taxonomic specificity differences. Coral *Fa* and *Ps* exhibited the highest overall similarity in enzyme abundance with relatively uniform distributions. This uniformity reflects the comprehensive metabolic capabilities of microbial communities in both corals, enabling them to address diverse substrate degradation and transformation demands within the coral habitat. This may be attributed to relatively consistent nutritional conditions in the habitats of these two corals (Zhou et al., 2021). The abundance of enzyme 2.7.11.1 in coral *Fs* was significantly higher than that of other enzyme classes, while the abundances of other enzymes were relatively low and evenly distributed. This characteristic suggests that enzyme 2.7.11.1 may serve as a key functional enzyme enabling the microorganisms in coral *Fs* to adapt to their specific habitat. It indicates that the habitat of coral *Fs* samples may be subject to specific environmental stresses, such as nutrient limitation, prompting microorganisms to enhance their environmental adaptability by enriching this enzyme. Coral *Md* exhibits the richest enzymatic functional diversity, with multiple enzymes showing high abundance levels that significantly differ from the other three groups. This high diversity may be linked to coral *Md*′s high species diversity (Figure 9): diverse microbial communities provide the foundation for the richness of enzymatic functions.

The four corals exhibited highly consistent characteristics across major functional categories (Figure 9), with metabolic functions accounting for the highest proportion by far. Environmental information processing and genetic information processing functions followed in prominence, while human disease and biological system functions accounted for an extremely low proportion. This distribution pattern reflects that the function of coral symbiotic microorganisms is to participate in material cycling and energy flow within the coral habitat. The low proportion of human disease and biological system functions indicates that the bacterial communities in the four sample groups primarily serve symbiotic and interactive roles with their coral hosts, rather than pathogenic functions. This aligns with the typical characteristics of healthy coral symbiotic microbial communities (Vilela et al., 2021), and provides a reference for subsequent investigations into the relationship between coral health status and microbial functions.

Meanwhile, each coral species exhibits distinct functional characteristics. The *Md* group’s functional profile differs significantly from the other three corals (Figure 9), likely due to its dominance by the phylum Firmicutes and higher fermentation capacity. This suggests an adaptation of its microbial community to relatively hypoxic or organic-enriched microenvironments. The *Fs* group exhibits higher potential in hydrocarbon degradation and related functions, likely associated with the absolute dominance of the genus *Ruegeria* within its microbiome, reflecting the specific adaptation of this microbial community toward degrading complex organic compounds. Additionally, the functional patterns of *Fa* and Fs groups are relatively similar, indicating that both may inhabit environments with comparable physicochemical properties where their microbial communities fulfill analogous ecological roles. These communities also exhibit functional distributions related to nitrogen and sulfur cycling, as well as photosynthesis, across different samples. These functions collectively contribute to nutrient cycling and energy replenishment in the host, providing a reference for subsequent investigations into the relationship between coral health status and microbial functions.

This study conducted a predictive analysis of the microbial community functions of four species of corals in the South China Sea. The results indicated that although the bacterial community compositions of different corals varied, their core functional categories, such as amino acid transport and metabolism, energy production and conversion, etc., exhibited highly conserved characteristics. This finding is consistent with previous research and further supports the view that coral symbiotic microbial communities may generally possess functional redundancy and ecological niche conservation.

This observation is supported by prior research. Gong et al. (2020) found that the core metabolic functional profiles of multiple coral species in the South China Sea were highly conserved, despite variations in geographical distribution and host species. Similarly, Zhou et al. (2021) reported significant functional conservation in the enzyme activity patterns of bacterial communities in *Orbicella annularis* corals from the Caribbean Sea. Varasteh et al. (2021) also noted that two phylogenetically distinct coral-symbiotic bacterial taxa maintained conserved pigment metabolism functions.

Notably, the microbiome of *Montipora digitata* diverged markedly from the coral norm, being dominated by Firmicutes rather than Proteobacteria. This suggests distinct host-specific or microenvironmental adaptation strategies. Despite this compositional shift, the functional profile of this community across Clusters of Orthologous Genes (COG) categories remained closely aligned with those of the other three coral species. This functional concordance demonstrates that variation in microbial composition does not necessarily disrupt the conservation of core metabolic processes within the holobiont.

## 5 Conclusion

In summary, based on previous studies, this study systematically reveals that the symbiotic bacterial communities of four coral species in the South China Sea exhibit significant compositional differences yet demonstrate highly conserved core metabolic functions. Through high-throughput sequencing and functional prediction analysis of four coral samples—*Fa*, *Fs*, *Md*, and *Ps*—a total of 23 phyla and 250 genera of bacterial taxa were identified. The *Md* group exhibited the highest microbial diversity, whereas the *Fs* group showed the lowest. Despite significant differences in community structure, both COG and KEGG functional analyses indicate that the four major coral microbial communities exhibit high consistency in core functional categories, indicating functional redundancy and niche conservation. It should be noted that functional predictions are based on bioinformatics methods and have not yet been experimentally validated. Therefore, caution should be exercised when inferring specific metabolic pathways. Future research should integrate metagenomic, metatranscriptomic, and culture-based experiments to further validate the specific functions of core microbial communities and their underlying mechanisms in coral health and environmental adaptation. This will provide more reliable theoretical foundations for coral reef ecological restoration and early warning systems for coral bleaching.

## Data Availability

Raw sequencing data presented in this study have been deposited in the NCBI Sequence Read Archive (SRA) repository under accession number PRJNA1370445.
