# Supplementary material for: Composition, diversity and functional potential of bacterial community in four stony coral species from the South China Sea
Source: Front Microbiol. 2026 Feb 10;17:1759094. doi: 10.3389/fmicb.2026.1759094 (PMC12929457; doi:10.3389/fmicb.2026.1759094)
Supplement: Supplementary file 1 [file Table_1.DOCX]

Table s1. The relative abundances of each sample at the phylum level.

| Phylum ID | *Fa* | *Fs* | *Md* | *Ps* |
| --- | --- | --- | --- | --- |
| Proteobacteria | 49.02±0.0878 | 56.72±0.0546 | 59.07±0.0659 | 62.19±0.0127 |
| Planctomycetota | 16.55±0.0357 | 12.26±0.0412 | 12.47±0.0423 | 9.3±0.0431 |
| Bacteroidota | 13.52±0.0368 | 17.66±0.0524 | 19.04±0.034 | 21.6±0.045 |
| Firmicutes | 2.1±0.0038 | 2±0.0056 | 1.56±0.008 | 1.2±0.0029 |
| Actinobacteriota | 5.75±0.0306 | 3.52±0.0082 | 2.95±0.0106 | 2.46±0.003 |
| d__Bacteria;k__norank_d__Bacteria;p__unclassified | 6.48±0.0218 | 4.59±0.0442 | 1.7±0.0265 | 0.22±0.0011 |
| Cyanobacteria | 1.75±0.0243 | 0.64±0.0052 | 0.72±0.0044 | 0.84±0.0038 |
| Acidobacteriota | 0.8±0.0085 | 0.25±0.0011 | 0.18±0.0007 | 0.12±0.0002 |
| Dadabacteria | 0.52±0.007 | 0.09±0.0006 | 0.1±0.0006 | 0.07±0.0001 |
| Chloroflexi | 0.63±0.0061 | 0.23±0.0008 | 0.22±0.0009 | 0.17±0.0002 |
| others | 2.89±0.0109 | 2.03±0.0071 | 1.99±0.0072 | 1.83±0.0046 |

*Fa*, *Favites abdita*; *Fs*, *Favia speciosa*; *Md, Montipora digitata*; *Ps*, *Porites solida*.

Table s2.The relative abundances of each sample at the genus level

| Genus ID | *Fa* | *Fs* | *Md* | *Ps* |
| --- | --- | --- | --- | --- |
| *Ruegeria* | 20.73±0.0829 | 23.13±0.0417 | 27.62±0.1098 | 29.26±0.1069 |
| *Flavobacteriaceae* | 2.88±0.0174 | 1.64±0.0042 | 1.56±0.0039 | 1.24±0.0027 |
| *Paramaledivibacter* | 0.11±0.0007 | 0.1±0.0007 | 0.11±0.0006 | 0.07±0.0001 |
| *Blastopirellula* | 3.09±0.0173 | 1.84±0.0051 | 2.27±0.0091 | 1.87±0.0111 |
| *unclassified_f__Rhodobacteraceae* | 1.92±0.0084 | 8.97±0.1141 | 9.01±0.1138 | 9.5±0.1096 |
| *g__unclassified_k__norank_d__Bacteria* | 6.48±0.0218 | 4.59±0.0442 | 1.7±0.0265 | 0.22±0.0011 |
| *Mycobacterium* | 2.46±0.0122 | 1.73±0.0005 | 1.45±0.0044 | 1.47±0.0046 |
| *Pir4_lineage* | 1.65±0.007 | 1.02±0.0043 | 0.94±0.0041 | 0.68±0.0021 |
| *Tenacibaculum* | 1.82±0.0138 | 6.66±0.0704 | 6.74±0.0696 | 7.46±0.0646 |
| *Woeseia* | 1.79±0.0293 | 0.1±0.0004 | 0.14±0.0003 | 0.13±0.0004 |
| *g__norank_f__Rubinisphaeraceae* | 1.24±0.0068 | 0.72±0.0024 | 0.78±0.0028 | 0.59±0.0032 |
| *Fuerstia* | 3.49±0.0203 | 3.81±0.0149 | 3.72±0.0144 | 2.54±0.0145 |
| *g__unclassified_o__Flavobacteriales* | 1.45±0.0157 | 1.93±0.0116 | 3.14±0.015 | 5.16±0.0386 |
| *g__norank_f__Methyloligellaceae* | 1.87±0.0021 | 1.47±0.009 | 1.12±0.0082 | 0.63±0.0025 |
| *g__unclassified_c__Alphaproteobacteria* | 1.46±0.0115 | 0.77±0.0013 | 0.77±0.0012 | 0.68±0.0025 |
| *g__norank_f__norank_o__Planctomycetales* | 0.81±0.0068 | 0.36±0.001 | 0.31±0.001 | 0.22±0.0006 |
| *g__unclassified_f__Rhizobiaceae* | 1.02±0.0014 | 0.93±0.0007 | 1.12±0.003 | 1.01±0.0041 |
| *g__norank_f__norank_o__Chloroplast* | 1.47±0.0219 | 0.55±0.006 | 0.65±0.0049 | 0.79±0.0041 |
| *Rubripirellula* | 1.31±0.0049 | 1.35±0.0043 | 1.43±0.0047 | 1.11±0.0047 |
| *Methyloceanibacter* | 0.61±0.0025 | 0.4±0.0025 | 0.39±0.0025 | 0.29±0.001 |
| *g__unclassified_f__Hungateiclostridiaceae* | 0.02±0.0001 | 0.02±0.0001 | 0.02±0.0001 | 0.01±0.0001 |
| *Cohaesibacter* | 0.31±0.0022 | 1.83±0.0242 | 2.12±0.0222 | 4.14±0.0256 |
| *g__norank_f__norank_o__Actinomarinales* | 0.66±0.0074 | 0.16±0.0012 | 0.12±0.0009 | 0.07±0.0004 |
| *Bythopirellula* | 0.69±0.0027 | 0.49±0.0014 | 0.58±0.0018 | 0.46±0.0023 |
| *Actibacterium* | 0.64±0.0023 | 0.68±0.0016 | 1±0.007 | 2.4±0.0222 |
| *g__norank_f__Rhizobiaceae* | 1.67±0.0093 | 1.88±0.0058 | 1.69±0.004 | 1.24±0.0058 |
| *g__unclassified_c__Gammaproteobacteria* | 0.89±0.0014 | 0.78±0.0022 | 0.73±0.0013 | 0.77±0.0015 |
| *Muricauda* | 1.59±0.009 | 1.92±0.0044 | 1.91±0.0042 | 1.71±0.0066 |
| *Rhodopirellula* | 0.6±0.0015 | 0.42±0.0018 | 0.34±0.002 | 0.23±0.0002 |
| *g__norank_f__norank_o__Dadabacteriales* | 0.52±0.007 | 0.09±0.0006 | 0.1±0.0006 | 0.07±0.0001 |
| *Filomicrobium* | 0.71±0.0025 | 0.46±0.0027 | 0.42±0.0028 | 0.25±0.0008 |
| *Crassaminicella* | 0.05±0.0004 | 0.05±0.0004 | 0.05±0.0004 | 0.03±0 |
| *g__norank_f__Kiloniellaceae* | 0.44±0.0036 | 0.19±0.0007 | 0.16±0.0007 | 0.12±0.0002 |
| *g__unclassified_f__Pirellulaceae* | 0.58±0.0046 | 0.25±0.001 | 0.2±0.0008 | 0.16±0.0002 |
| *Leisingera* | 0.48±0.004 | 0.22±0.0013 | 0.12±0.0004 | 0.13±0.0005 |
| *Planctomicrobium* | 0.55±0.0022 | 0.39±0.0012 | 0.39±0.0012 | 0.31±0.0003 |
| *g__norank_f__Cyclobacteriaceae* | 0.35±0.0016 | 0.24±0.0018 | 0.26±0.0018 | 0.17±0.0006 |
| *Actibacter* | 0.79±0.0075 | 1.13±0.005 | 1.27±0.0031 | 1.26±0.003 |
| *Coxiella* | 0.46±0.0025 | 1.44±0.015 | 1.37±0.0155 | 1.33±0.0158 |
| *Labrenzia* | 0.3±0.0007 | 0.29±0.0009 | 0.49±0.0034 | 1.16±0.0112 |
| *Microbulbifer* | 0.61±0.0044 | 0.5±0.0054 | 0.45±0.0058 | 0.11±0.0001 |
| *g__norank_f__Rhodothermaceae* | 0.34±0.0058 | 0±0 | 0.01±0 | 0.01±0.0001 |
| *g__norank_f__Anaerovoracaceae* | 0.01±0.0001 | 0.01±0.0001 | 0.01±0.0001 | 0.01±0 |
| *g__unclassified_o__Rhizobiales* | 0.22±0.0006 | 0.17±0.0005 | 0.26±0.002 | 0.82±0.009 |
| *Ilumatobacter* | 0.68±0.0041 | 0.35±0.0022 | 0.28±0.0025 | 0.14±0.0001 |
| *Jejudonia* | 1.09±0.0087 | 1.25±0.0059 | 0.92±0.0047 | 0.62±0.001 |
| *Anderseniella* | 0.27±0.0017 | 0.2±0.0005 | 0.23±0.0008 | 0.22±0.0009 |
| *Legionella* | 1.42±0.0191 | 1.43±0.0191 | 0.28±0.0026 | 0.17±0.0007 |
| *Spiroplasma* | 0±0 | 0±0 | 0±0 | 0±0 |
| *Seonamhaeicola* | 0.01±0.0001 | 0.01±0.0002 | 0.17±0.0026 | 0.78±0.0095 |
| *Lutibacter* | 0±0 | 0.01±0.0001 | 0.05±0.0006 | 0.46±0.0069 |
| others | 21.29±0.0569 | 16.04±0.0423 | 15.05±0.045 | 12.51±0.0078 |

*Fa*, *Favites abdita*; *Fs*, *Favia speciosa*; *Md, Montipora digitata*; *Ps*, *Porites solida*.
